# Therapeutic applications of IgY in common canine and feline viral diseases

**DOI:** 10.1016/j.vas.2025.100534

**Published:** 2025-10-28

**Authors:** Kamyar Madani, Nima Neyestani, Jalil Mehrzad, Darioush Shirani, Niloofar Zarifian

**Affiliations:** a Department of Microbiology and Immunology, Faculty of Veterinary Medicine, University of Tehran, Tehran, Iran; b Department of Internal Medicine, Faculty of Veterinary Medicine, University of Tehran, Tehran, Iran

**Keywords:** Immunoglobulin Y, IgY, CPV-2, FeLV, FIV, FIP, Immunotherapy

## Abstract

**Background:**

Immunoglobulin yolk (IgY) is the major antibody in birds whose immunotherapeutic potential has been investigated in canine and feline viral diseases. Management currently focuses on supportive care (often insufficient) or prevention using vaccines (slow to develop). IgY is developed against a wide range of antigens, and commercial formulations also exist. Several therapeutic trials and case reports examine IgY as main or adjunctive treatment in feline and canine viral diseases.

**Objectives:**

To map and summarize the available evidence on IgY-based immunotherapy for viral infections of dogs and cats and assess its reported benefits and shortcomings in a scoping review following the PRISMA-ScR guidelines.

**Eligibility criteria:**

Original English-language studies, focusing on the application of IgY in treating canine and feline viral diseases.

**Sources of evidence:**

Six databases (Web of Science, Scopus, ScienceDirect, Google Scholar, PubMed, ResearchGate) were searched in October 2024 using combinations of keywords related to IgY, small animals and therapy.

**Charting methods:**

Data on study design, treatment protocol/dosage/outcome and OCEBM level of evidence score were extracted.

**Results:**

In total, 14 studies met inclusion criteria, including 7 clinical trials, 3 case series and 4 case reports; ten investigated CPV-2, while four examined feline viruses (FIV/FeLV/FIP). Most were small non-randomized clinical trials. IgY was administered orally, intravenously, and intranasally. Higher doses generally produced stronger effects. Feline studies used non-specific IgY with limited therapeutical benefit.

**Conclusions:**

IgY-based immunotherapy appeared safe, accelerated recovery and reduced mortality in most studies, but current evidence in feline viral infections is limited and non-specific. Further pathogen-specific trials are required.

## Highlights

• This is the first scoping review of IgY immunotherapy in canine and feline viral infections.
• Fourteen studies were identified: 10 on CPV-2, 4 on feline viruses (FIV/FeLV/FIP).
• Most studies were small, non-randomized clinical trial of moderate evidence level.
• Canine studies (CPV-2) consistently reported faster recovery rates and enhanced hematological values, and even prophylaxis.
• Feline studies used a non-specific IgY preparation with limited therapeutic benefit.

## 1 Introduction

### 1.1 Objectives and rationale for conducting scoping review

Immunoglobulin yolk (IgY) is the main class of antibodies present in birds, which is transported from the serum to egg yolks and stored in large quantities. Due to the binding ability of antibodies to specific targets, they are widely used in research, diagnosis and therapy. Most of these antibodies are produced in mammals (immunoglobulin G [IgG]), but using polyclonal IgY instead of mammalian IgG has several advantages: 1) no need for animal bleeding, 2) short time for preparation and production, 3) high yield of antibody production (El-Kafrawy et al., 2023; Lanzarini et al., 2018). In recent years, immunotherapy (including IgY therapy) as a new modality for treating refractory or complicated cases of canine and feline infectious diseases has gained popularity. The number of IgY patent applications has increased dramatically after 2010, with 77 patents filed in 2021. From 2010 to 2022, 56 % of IgY-based therapy patent applications filed were regarding products used as therapeutics and prophylactics, and 41 % of biotherapeutic products, were branded for veterinary use (Yakhkeshi et al., 2022).

Viral diseases are commonly either treated with supportive, non-specific therapy (and sometimes specific antiviral drugs), or prevented using vaccines. Supportive therapy is not effective enough in many cases, and developing antiviral drugs and vaccines is often costly and time-consuming. IgY may provide a low cost, safe, and fast (5–8 weeks, compared to years of research needed for vaccines and antiviral drugs) solution for prophylaxis and therapeutic development against many pathogens (Lee et al., 2021). Antibiotic resistant bacteria are a growing threat, brought upon by inappropriate use of antibiotics in healthcare leading to antimicrobial resistance (AMR). Passive immunization through oral, nasal and topical administration of IgY has been effective in treating bacterial infection in animals and humans. IgY can be developed against a wide range of specific antigens, including bacteria, viruses and even bacterial enzymes giving rise to AMR. IgY can also be used in immunocompromised individuals, for whom conventional treatment or vaccination is not effective (El-Kafrawy et al., 2023).

The main question this scoping review aims to answer is what evidence exists regarding the use of IgY-based immunotherapy as treatment, prophylaxis or adjunctive therapy for viral diseases in dogs and cats. A scoping review methodology was selected because of the limited number of available trials and the heterogeneity of study designs, which made a systematic review or meta-analysis infeasible. As IgY-based immunotherapy is still a developing field of study, this approach allowed us to map the existing evidence, summarize therapeutic applications/outcomes, and identify key knowledge gaps. Our aim was to provide an overview of the current literature regarding IgY-based immunotherapy in canine and feline viral infections, assess this method’s therapeutic potentials, reported benefits and shortcomings, and highlight areas where further research is needed, including what pathogens should be prioritized for future study.

### 1.2 IgY structure and characteristics

**Fig. 1:**
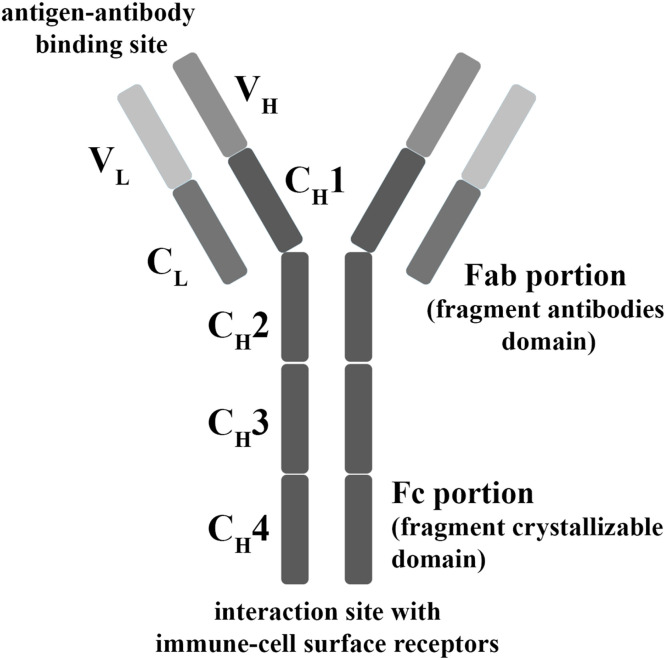
A schematic of the structure of IgY; V_L_: variable region in the light chain; V_H_: variable region in the heavy chain; C_L_: constant region in the light chain; C_H_1–4: constant regions in the heavy chain.

The IgY molecule is structurally similar to IgG, as it is its evolutionary the precursor. IgY has two heavy chains (H) and two light chains (L), each with a molecular weight of 70 and 25 kDa respectively, amounting to a total weight of 180 kDa. Each chain in the structure is formed by a N-terminal with variable domains (V) and a C-terminal consisting of constant domains (C). The light chains have one constant region (CL) and one variable (VL), while the heavy chain consists of one variable region (VH), and four constant regions (CH1–4). IgY is divided into two main portions, the Fab portion (fragment antibodies domain, which includes the antigen-antibody binding site), and the Fc portion (fragment crystallizable domain, which interacts with immune cell surface receptors and has biological effector functions). IgY, unlike IgG, does not include a hinge region connecting the Fab and Fc portions, which makes it less flexible and more resistant to proteolytic degradation (da Silva et al., 2021; Pereira et al., 2019). A schematic of the structure of IgY is demonstrated in Fig. 1.

### 1.3 IgY production and purification

At least 4 factors influence antibody production in the immunized hen: 1) antigen dose and molecular weight, 2) type of adjuvant, 3) route of application, and 4) circumstances of the hen (age, breed, keeping conditions). Several antigen types are used to produce IgY in birds; they include complex antigens (virus, bacteria, etc.) and single antigens (proteins, polysaccharides, peptides and nucleic acids). Optimal antigen dose must be tested experimentally, but it is common to use 10–100 µg (1 mg max) of antigen in 0.5–1 mL of volume, injected in two or three sites of a hen between six to eight weeks of age, and then repeated after 4–8 weeks. The titer of IgY must be assessed 14 days after the last immunization, and if it decreases, further inoculations are needed during the laying period (Pereira et al., 2019; Schade et al., 2005).

The production of IgY is facilitated by using adjuvants which stimulate B-cells. This stimulation is independent of the antigen but improves the magnitude of the immune response and antibody production non-specifically. The type of adjuvant used depends on the strength of the immunogen. Freund’s complete adjuvant (FCA) is the most effective adjuvant but is also a very potent tissue-damaging agent. Freund’s incomplete adjuvant (FIA) is less effective but also results in lower side-effects. It is recommended that either FCA be only used in weakly immunogenic antigens, or only used in the first inoculation, with subsequent inoculations preformed using FIA. The most common route of antigen administration in hens is intramuscular (IM) injections into the breast muscle. Subcutaneous (SC) injections into the neck are difficult and causes more distress in the animal, though it provokes a higher antibody titer. The intravenous (IV) route can only be used without adjuvant and IV injections must be done very slowly (500 µL over 15 min) to avoid anaphylactic shock. Oral (PO) administration of antigens is also possible as a non-invasive method (Pereira et al., 2019; Schade et al., 2005).

**Fig. 2:**
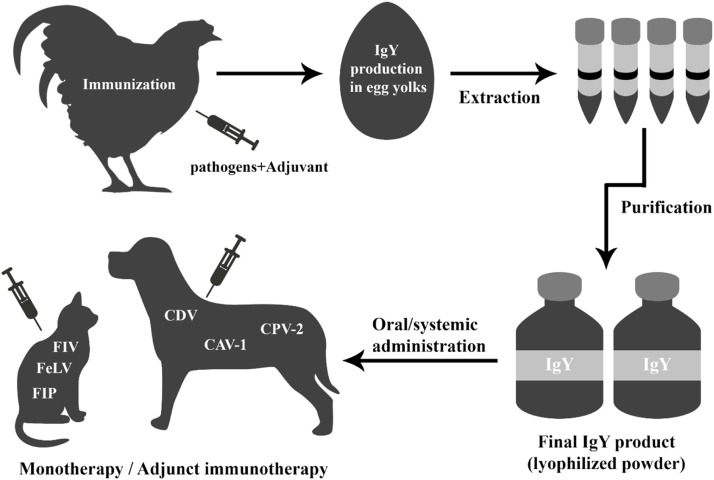
A summary of the IgY production pipeline.

After producing antibodies in the egg yolk, the IgY needs to be extracted or purified. The choice of the extraction method depends on the scale of IgY purification needed (laboratory vs. industrial), cost effectiveness, and availability. In general, these methods are divided into three main groups: 1) precipitation methods: involving ammonium or sodium sulphate, polyethyleneglycol (PEG), caprylic acid, and carrageenin, 2) chromatographic methods: based on affinity, ion exchange, hydrophobic interaction, thiophilic interaction, and gel-filtration, and 3) ultrafiltration. The PEG precipitation method is one of the most common and effective methods currently used (Schade et al., 2005). A summary of the IgY production pipeline is shown in Fig. 2.

## 2 Materials and methods

### 2.1 Study design and review protocol

This scoping review was conducted following the PRISMA extension for scoping reviews (PRISMA-ScR) guidelines (Tricco et al., 2018). The search methodology, assessment for eligibility, and data extraction steps were carried out as outlined in the protocol. As stated before, because IgY-based immunotherapy remains a developing field with relatively few studies on the subject, a limited number of randomized clinical trials, and considerable heterogeneity in study designs and outcome measurement methods, a formal meta-analysis or systematic review was not feasible. Instead, a scoping review approach was chosen to map and summarize the available literature, describe study designs and characteristics, treatment protocols/outcomes, and identify knowledge gaps, rather than to conduct a detailed risk-of-bias assessment.

### 2.2 Eligibility criteria

Eligible articles had to be original research, published in English, and provide full-text availability. Studies were required to focus on the application of IgY therapy in treating viral diseases in dogs and cats. Any research involving the therapeutic use of IgY against specific pathogens in canine and feline infections was considered eligible. In contrast, studies that solely focused on other types of therapies or species were deemed ineligible, as the aim of this review was to explore IgY-based approaches in canine and feline health.

### 2.3 Information sources and literature search

The literature search was conducted at the end of October 2024 across six databases to identify potentially relevant articles: 1) Web of Science (https://www.webofscience.com/), 2) Scopus (https://www.scopus.com/), 3) ScienceDirect (https://www.sciencedirect.com/), 4) Google Scholar (https://scholar.google.com/), 5) PubMed (https://pubmed.ncbi.nlm.nih.gov/), 6) ResearchGate (https://www.researchgate.net/). These databases were selected based on their relevance to both veterinary medicine and immunotherapy.

The electronic search strategy was initially developed in Google Scholar and later adapted for the other databases. It utilized a combination of keywords related to IgY, small animals and therapy. The search terms included "IgY", “immunoglobulin Y”, "canine", "feline", "treatment" and "therapy." These terms were combined systematically to maximize relevant study retrieval (the resulting search string would look like the following: “IgY/Immunoglobulin Y” AND “Canine/Feline” AND “Treatment/Therapy”). Furthermore, this review included all available journal articles, conference papers, dissertations, case reports, and other scholarly publications, provided they were: 1) Written in English, and 2) Available in full text. There were no restrictions on the type of publication or the geographical origin of the study. Any article meeting these criteria was considered for further evaluation. The number of studies retrieved from each database using different combinations of keywords is shown in Table 1.

**Table 1:** Preliminary data regarding the number of retrieved articles from 6 databases using each combination of keywords. Each keyword was put in quotation marks (“”) and the word “AND” with all capital letters was used in between them.

1st keyword:	IgY				Immunoglobulin Y			
2nd keyword:	Canine		Feline		Canine		Feline	
3rd keyword:	Treatment	Therapy	Treatment	Therapy	Treatment	Therapy	Treatment	Therapy
Google Scholar	1530	943	534	328	278	200	117	83
Scopus	5	2	4	1	4	3	3	1
Springer	50	30	15	10	5	3	0	0
ScienceDirect	158	5	53	0	34	26	13	12
PubMed	1	1	2	2	0	0	1	0
Web of Science	2	2	4	1	1	2	3	1

### 2.4 Study selection and screening process

**Fig. 3:**
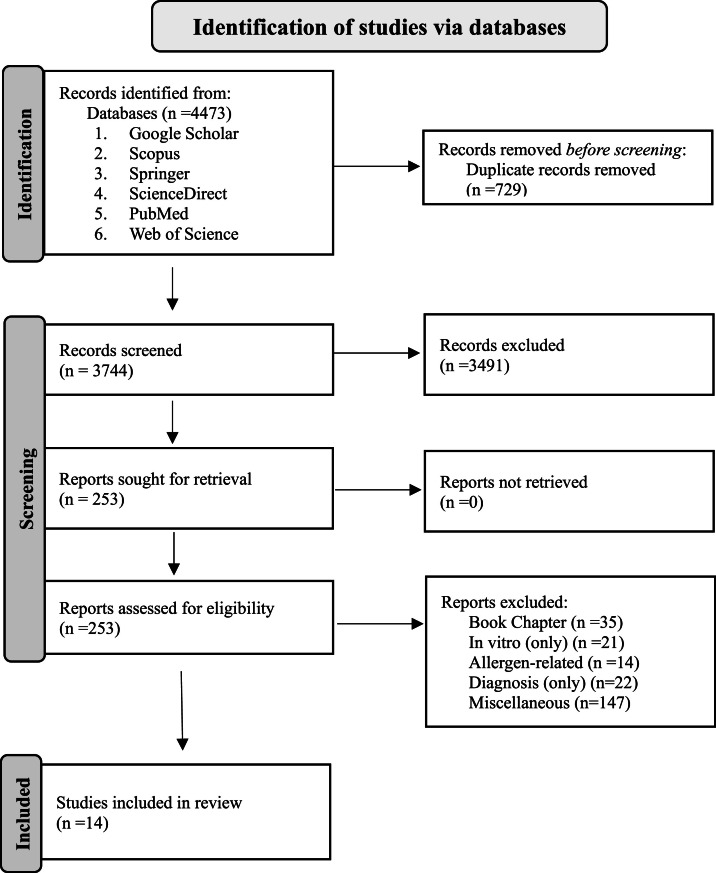
PRISMA 2020 flow diagram for systematic reviews used as inspiration for this review’s study design and search protocol (Page et al., 2021).

Hits from the literature search were uploaded into Mendeley for organization and duplicates were removed. The study selection involved two levels of screening:

1) Title and Abstract Screening: This initial stage aimed to ensure that all relevant literature was considered for full-text review. Two independent reviewers (NN and KM, under the supervision of JM and DS) screened titles, abstracts, and keywords using a predefined checklist, which included the following questions: 1) Was the title/abstract written in English? 2) Was the article available online? 3) Did the study involve the application of IgY in veterinary medicine? 4) Did the study focus on canine or feline diseases? Eligible responses were yes, no, or unclear. If both reviewers responded “no” to a criterion, the study was excluded. Disagreements were resolved by discussion between both reviewers in a meeting.
2) Full-Text Screening: Studies that passed the first screening phase were retrieved in full text and reviewed by both researchers. The following questions guided the second stage of screening: 1) Were the specific pathogens targeted by IgY therapy clearly identified? 2) Did the study include in vivo trials to evaluate the efficacy of IgY? 3) Were the results of the IgY therapy analyzed and reported? 4) Did the study focus on treating infectious diseases in dogs or cats? Studies that answered "yes" to all questions were categorized based on the type of pathogen and the specific infectious disease targeted. A “no” response to any question resulted in the exclusion of the study from the final review. A summary of the search protocol used in this review is provided in Fig. 3.

### 2.5 Levels of evidence scoring system and critical appraisal

In line with scoping review methodology, no formal risk-of-bias assessment was performed. The available evidence was highly heterogenous, consisting largely of small non-randomized trials, case series and case reports, which were not well-suited to standardized bias measurement tools. Instead, authors graded gathered articles based on the robustness of their research method using a modified version of the Levels of Evidence Table (OCEBM Levels of Evidence Working Group, 2011) published by the Oxford Centre for Evidence-Based Medicine. Each article was awarded a L_E_ (level of evidence) score from A (the highest) to D (the lowest) according to the following criteria: A) Randomized trial or observational study with dramatic effect, B) Non-randomized controlled cohort/follow-up study, C) Case-series, case-control studies, or historically controlled studies, D) Case report, Mechanism-based reasoning. This approach allowed for a qualitative indication of study strength and was considered appropriate given our objective of mapping the evidence base rather than performing a comprehensive meta-analysis.

### 2.6 Data charting process

Each study was carefully assessed and data items including their L_E_ score, study design (number of experimental and control groups, if applicable), treatment protocol/outcome and dosage (if specified) were gathered and compiled (by NZ). A summary of collected data items is available in Table 2.

**Table 2:** summary of the articles reviewed herein. LE: level of evidence; PO: oral; IV: intravenous; SID: once daily; BID: twice daily; PCR: polymerase chain reaction; HA: Hemagglutination assay; ELISA: enzyme-linked immunosorbent assay; ALT: alanine transaminase; AST: aspartate transferase; ALP: alkaline phosphatase; GGT: gamma-glutamyl transferase; PD50: 50 % protective dose; PCV: packed cell volume; PPIs: proton-pump inhibitors; NSAIDs: non-steroidal anti-inflammatory drugs.

Reference	L_E_	Virus	Experimental and control groups of Animals	Treatment Protocol	Outcome of IgY treatment
(Van Nguyen et al., 2006)	B	CPV-2	10 beagle dogs orally challenged with a strain of the virus	**Control** (*n* = 4): normal egg yolk **Group1** (*n* = 3): 2 g of IgY powder. **Group2** (*n* = 3): 0.5 g of IgY powder. *IgY was administered orally shortly after inoculation, before clinical signs.	**Control**: mild symptoms, such as vomiting, diarrhea, and weight loss. **Group1**: No symptoms were observed by day 16. Prophylaxis achieved. **Group2**: 2 had clinical sings but less severe compared to controls. *Shorter duration of viral shedding in both treatment groups compared to controls.
(Suartini et al., 2014)	B	CPV-2	16 puppies between the ages of 2 and 4 months	**Control (-)**: not infected + untreated **Control (+)**: infected + untreated **Group1**: low dose of IV IgY (1000 PD_50_) **Group2**: high dose of IV IgY (10,000PD_50_)	The recovery rates for the dogs treated with 1000 and 10,000 PD were 25 % and 100 %, respectively.
(Naveenkumar et al., 2019)	D	CPV-2	5 months old, male, Rottweiler with hemorrhagic diarrhea, vomiting and inappetence. CPV-2 confirmed via molecular methods.	Fluid therapy + broad spectrum antibiotics + antiprotozoal + gastric protectant + antiemetics + Parvocure IgY Tablet SID up to six days.	IgY-mediated neutralization of viral load reduced damage and other complications.
(Chethan, 2020)	A	CPV-2	60 CPV-2 (+) dogs randomly assigned to 10 treatment groups, 6 CPV-2 (-) dogs	**I** (Control, *n* = 6): Placebo. **II** (*n* = 6): Supportive treatment (ST) including IV fluids+ antibiotics+ antiemetics ± PPIs+ haemostatic+ NSAIDs. **III** (*n* = 6): ST+ *N*-acetylcysteine. **IV** (*n* = 6): ST+ Resveratrol. **V** (*n* = 6): ST+ Coenzyme Q10. **VI** (*n* = 6): ST+ Ascorbic acid. **VII** (*n* = 6): ST+ IgY. **VIII** (*n* = 6): ST+ *N*-acetylcysteine+ IgY. **IX** (*n* = 6): ST+ Resveratrol+ IgY. **X** (*n* = 6): ST+ Coenzyme Q10+ IgY. **XI** (*n* = 6): ST+ Ascorbic acid+ IgY.	**IgY groups:** 1) enhanced leukocyte and neutrophil counts. 2) marked improvement in mean concentrations of total protein, albumin, and globulin. 3) decreased mean concentration of ceruloplasmin. 4) earlier palliation of symptoms. 5) lower clinical scores, fecal viral load, and cortisol level. 6) restoration of trace mineral homeostasis.
(Rudra Paul, 2022)	B	CPV-2	6 CPV (-) dogs and 16 mildly, 18 moderately and 17 severely affected CPV-2 (+) dogs	**Control** (*n* = 6): Placebo (Sterile water), PO, BID × 7 days **Mild-ST** (*n* = 10): Supportive treatment (ST) for 7 days **Mild-ST+IgY** (*n* = 6): ST for 7 days+ IV IgY for 3 days **Moderate-ST** (*n* = 11): ST for 7 days **Moderate-ST+IgY** (*n* = 7): ST for 7 days+ IV IgY for 3 days **Severe-ST** (*n* = 10): ST for 7 days **Severe-ST+IgY** (*n* = 7): ST for 7 days+ IV IgY for 3 days	**IgY groups:** 1) higher mean hemoglobin, PCV, total protein, albumin, globulin. 2) lower C-reactive protein, ceruloplasmin, clinical score, viral fecal titer values. 3) higher total leukocyte, neutrophil counts. 4) IgY showed better efficacy in mild and moderate groups compared to the severe group.
(Naveenkumar et al., 2023)	B	CPV-2	60 naturally infected dogs (confirmed by PCR)	**Control** (*n* = 30): Standard treatment (crystalloids + antibiotics + gastric protectant + antiemetics). **IgY-treated** (*n* = 30): Standard treatment + IgY tablets (Parvoguard™) PO	Clinical scores returned to normal within 9 days, while dogs in the control group needed an additional 2 days of treatment. 83.33 % survival rate with IgY, only 60 % survival rate in controls.
(Deka et al., 2024; Rishikesavan et al., 2021)	D	CPV-2	Each treated a German shepherd puppy (3 and 4 months old, respectively) with CPV-2 infection confirmed by HA test	Canglob-P® (0.4 ml/Kg IV) for 5 days + antibiotics + H2-blockers + antiemetics	Pups survived and their recovery was accelerated.
(Sudhakara Reddy et al., 2023)	C	CPV-2	6 dogs (between 2 and 6 months) confirmed to have CPV-2 infection (using an immunochromatographic fecal antigen test)	IV Canglob- P® to in addition to routine supportive therapy (antibiotics + H2-blockers + antiemetics + *B* complex vitamins).	Immunotherapy had significant effect on improving hematological indices such as hemoglobin, PCV, total erythrocyte count, and total leukocyte, neutrophil, lymphocyte counts.
(Ali et al., 2024)	B	CPV-2, CAV-1, CDV	8 dogs aged from 4 to 9 months with CPV-2 (*n* = 4), CAV-1 (*n* = 2), and CDV (*n* = 2) infections	**Control** (*n* = 4): maintenance therapy **IgY-treated** (*n* = 4): maintenance therapy + 150 ml of IgY solution PO BID for 5 days, on an empty stomach + rinsed nasally twice a day by pipette with 10 ml of IgY.	The experimental group entered recovery stage on day 5 and 6 while dogs in the control group recovered on days 7 and 8.
(Supeanu et al., 2015)	C	FIV, FeLV	8 FIV (+) and 4 FeLV (+) cats (ELISA and PCR used to confirm FeLV, ELISA and Western Immunobloting used to confirm FIV)	Treated with a non-specific IgY solution obtained by immunizing chickens against a mixture of several inactivated bacterial and fungal strains administered daily for 10 days, at a dosage of 10 mg, PO.	**In FIV infected cats:** improved quality of life and neutrophil, platelet, lymphocyte, and erythrocyte counts; improved ALP and GGT. **In FeLV infected cats:** improved neutrophil and lymphocyte counts as well as ALP.
(Supeanu et al., 2016)	C	FIV	8 FIV (+) cats over a 5 months period	Same as Supeanu et al. (2015).	Leukocyte counts as well as albumin and globulin values (and their ratio) tend to evolve towards physiological values
(Supeanu et al., 2017)	D	FIP	a cat with effusive FIP	Same as Supeanu et al. (2015).	Prolonged survival time of 13 days after starting treatment, which was above the average of 8–9 days previously reported.
(Supeanu et al., 2020)	B	FIV	10 FIV (+) and 10 FIV (-) cats	40-day study, IgY treatment in first 20 days. Treated with the same IgY product as Supeanu et al. (2015) in a concentration of 200 mg/100 mL of solution. 10 mg IgY SID, 4 mL oral solution, for 20 days.	1) Improvement in symptoms on D20, clinical signs relapsed from D20 to D40 2) Increased ALT and AST by D20, D0 values on D40. 3) Improvement in hematological values 4) decreased pro-inflammatory cytokines, inconsistent effects on anti-inflammatory cytokines.

## 3 Results

A total of 14 studies were included based on the predefined eligibility criteria. Bellow, they are presented as a narrative synthesis, structured by pathogen, to provide a descriptive overview of study characteristics, evidence strength and findings of available literature.

### 3.1 Canine parvovirus type 2 (CPV-2) and other canine viral diseases

Canine parvovirus type 2 (CPV-2) is a highly contagious pathogen of dogs which was first recognized in the late 1970s and remains a major cause of morbidity and mortality in young, unvaccinated animals (Nguyen & Danh, 2024; Tuteja et al., 2022). The virus likely emerged from feline panleukopenia virus by VP2 mutations, enabling adaptation to canine hosts (Tuteja et al., 2022). Transmission is predominantly fecal–oral. The virus persists in the environment for extended periods and resists many disinfectants (Martins et al., 2024). CPV-2 targets rapidly dividing cells, particularly intestinal crypt epithelium, bone marrow, and lymphoid tissues, causing villous atrophy, leukopenia, and, in some neonatal cases, myocarditis (Mylonakis et al., 2016; Prittie, 2004). Clinical signs include vomiting, hemorrhagic diarrhea, lethargy, and dehydration. Supportive therapy, including aggressive fluid resuscitation, antiemetics, and, when indicated, antimicrobials, remains the mainstay of treatment (Mylonakis et al., 2016). Vaccination is the most effective preventive measure, though surveillance of emerging variants is vital to maintain vaccine efficacy (Yadav et al., 2024).

A total of 10 studies evaluated the effects of IgY-based immunotherapy in CPV-2 infections: 1 randomized clinical trial (RCT), 5 non-randomized controlled clinical studies, 3 case reports, and 1 case series. Sample sizes were generally small, with only 3 studies enrolling >15 dogs while sample size in the rest was ≤10. Most investigations used IgY as an adjunct to standard supportive care (intravenous fluids, antibiotics, antiemetics and gastric protectants, with some variations per study or case), while 2 trials used IgY as their main treatment method. 2 non-randomized controlled trials assessed the effect of IgY dosage on clinical signs and recovery rate and both suggested greater benefits at higher dosages (Suartini et al., 2014; Van Nguyen et al., 2006), while another investigated the effects of disease severity on treatment outcome and found IgY immunotherapy more efficacious in mild or moderately ill dogs, compared to severe infections (Rudra Paul, 2022). Among the studies that reported their diagnostic confirmation method, PCR and immunochromatography tests were the most common.

Placebo groups were included in 2 studies (L_E_ scores: A-B) in addition to groups receiving standard supportive treatment, whereas 1 study incorporated a negative (not infected) and a positive (infected, not treated) control group. 4 studies produced their own IgY antibody solutions by immunizing hens with CPV-2 antigen, whereas 5 studies used commercial IgY formulations (2 in tablet form, 3 as liquid suspension). IgY administration method was intravenous (IV) in 5 studies and oral in the remainder. 3 studies (L_E_ scores: A-C) investigated the effects of IgY immunotherapy on hematological indices and reported enhanced leukocyte and neutrophil counts as well as higher mean hemoglobin, PCV and total protein levels. 3 studies (L_E_ scores: A-B) reported decreased fecal viral load in IgY treated dogs.

One non-randomized controlled trial was unique in investigating the effects of IgY against CPV-2, canine distemper virus (CDV) and canine adenovirus-1 (CAV-1) simultaneously, using IgY derived from hens immunized with a polyvalent vaccine which contained the antigens of all these viruses (Ali et al., 2024). Notably, this was the only trial to investigate intranasal administration of IgY in additions to the oral route. Despite being limited by a very small sample size (≤2 for each control and patient group) across all investigated diseases, the study reported faster recovery rates in all IgY treated dogs. Another non-RCT was unique as it was the only trial which administered oral IgY prophylactically, before the appearance of clinical symptoms. Notably, though the sample size was small, the study reported complete prophylactic inhibition of symptoms at high doses (Van Nguyen et al., 2006).

Overall, included studies reported faster recovery rates and alleviation of symptoms with no discernable side effects; However, aside from 2 non-randomized controlled trials and 1 RCT, the remainder of the studies were either limited by small sample sizes or a study design which scored low in OCEBM levels of evidence chart. Details of each study is summarized in Table 2.

### 3.2 Feline immunodeficiency virus (FIV), feline leukemia virus (FeLV) and Feline infectious peritonitis (FIP)

Feline Immunodeficiency Virus (FIV) and Feline Leukemia Virus (FeLV) are major retroviral pathogens of cats, differing in pathogenicity, epidemiology, and clinical impact (Hartmann, 2012). FeLV, is a gammaretrovirus which causes severe diseases such as leukemia and lymphoma, while FIV is a lentivirus similar to HIV, primarily inducing progressive immunodeficiency (Hosie et al., 2009; Jarrett & Neil, 2012). FIV mainly transmits via bites and uses CXCR4 as its primary receptor (Miyazawa, 2002). Risk factors for FIV include male gender, and outdoor roaming, while for FeLV, the absence of vaccination is the leading risk factor (Diesel et al., 2024; Yamamoto et al., 1989). FeLV infection is transmitted horizontally through close contact between cats (especially through contact with saliva) and vertically from infected queens to their kittens (Zdziennicka et al., 2023). Clinically, FIV often remains latent for years, later manifesting as chronic gingivostomatitis, weight loss, and opportunistic infections. FeLV tends to produce tumors, anemia, and profound immunosuppression (Collado et al., 2012; Hartmann, 2012). No specific treatment is available; management focuses on supportive care, transmission prevention, and FeLV vaccination (not for FIV) (Levy et al., 2008). Regular testing, controlled housing, and segregation of infected cats are needed for prevention (Hartmann & Hofmann-Lehmann, 2020).

Feline infectious peritonitis (FIP) is a fatal disease caused by mutation of feline coronavirus (FCoV) from the enteric form (FECV) to the macrophage tropic FIP virus (FIPV) which alters tissue tropism and pathogenicity (Kennedy, 2020; Kipar & Meli, 2014). It has worldwide distribution, especially in cats under two years and in multi-cat environments, with stress and overcrowding as key risk factors (Anwer et al., 2022; Tasker et al., 2023). Transmission is primarily fecal–oral, with high FCoV prevalence but sporadic progression to FIP (Wang et al., 2013). The disease manifests in effusive (wet) and non-effusive (dry) forms, presenting with fever, weight loss, and organ specific signs, including ocular and neurological involvement (Barr, 2008; Thayer et al., 2022). Its pathogenesis is immune-mediated vasculitis, with monocyte/macrophage activation and antibody dependent enhancement complicating immunity and vaccine development (Kipar & Meli, 2014; Solikhah et al., 2024). The most transformative advance has been antiviral therapy with GS-441,524 and remdesivir, achieving 80–90 % cure rates in early diagnosed cases (Gokalsing et al., 2025; Taylor et al., 2023). Protocols typically span 84 days, with shorter courses under investigation, and alternatives like molnupiravir and GC376 showing promise (Cerna, Knies et al., 2025, 2025). Preventive efforts center on hygiene, population control, and novel strategies such as stopping FCoV shedding to avert mutation (Addie et al., 2023).

A total of 4 studies evaluated IgY-based immunotherapy in treating feline viruses: 1 non-randomized controlled trial and 1 case series which focused solely on FIV, 1 case series which investigated FIV and FeLV and 1 case report which focused only on FIP. All identified studies were conducted by the same two researchers (T.D. Supeanu and A. Supeanu), although co-authors varied throughout publications. In every study, the IgY was administered orally, and acquired by immunizing hens against various bacterial and fungal antigens, not FIV/FeLV/FIP antigens, making the antibodies non-specific. All studies were limited in sample size (≤10 for patient groups) and most ranked low in OCEBM levels of evidence chart (L_E_ scores: B-D). There were no RCTs identified that investigated the effects of IgY on feline patients.

None of the studies incorporated placebo controls; only the non-randomized clinical trial included a comparator group, which lacked a positive control arm. Instead, in addition to FIV negative controls, the IgY treatment was withdrawn after 20 days, which led to reappearance of symptoms, suggesting treatment effect was dependent on continued IgY administration. This study was also the only one which conducted a cytokine evaluation and reported that IgY decreased pro-inflammatory cytokines, but its effects on anti-inflammatory cytokines were inconsistent (Supeanu et al., 2020). Reported outcomes in FIV cases included improved leukocyte, neutrophil and lymphocyte counts, as well as alleviated symptoms, better quality of life and enhanced hematological indices. In contrast, the single FIP case observed only a modest extension of survival time, just a few days beyond average expectation. In FeLV cases, improved neutrophil and lymphocyte counts were also reported. Overall, the studies reported mixed results, but no adverse reactions to IgY were reported and its general effects on patient health was concluded as positive. Details of the studies can be found in Table 2.

## 4 Discussion

### 4.1 Overall state of the evidence

Across most studies collected and reviewed herein, IgY-based immunotherapy led to faster recovery rates, lower mortality, enhanced leukocyte counts and hematological factors, and overall palliation of clinical symptoms. Evidence for the therapeutic effects of IgY in canine and feline viral infections was strongest in the case of CPV-2 infections, with over 10 out of 14 studies focusing on this pathogen.

### 4.2 Limitations of the scoping review process

There were several limitations in the process of this scoping review that must be addressed. First, as stated, the very decision to conduct a scoping review was itself shaped by several limitations of the evidence base, such as the scarcity of research on the matter, the heterogeneity of study designs, limited number of robust and large trials, and the developing nature of the field, which precluded a formal systematic review or meta-analysis. As a result, the findings of this review should be considered as a descriptive mapping of the available literature surrounding the subject, rather than a definitive synthesis of efficacy. Second, consistent with the methodology of scoping reviews, we did not apply a formal risk-of-bias measurement tool; instead, studies were qualitatively appraised using a modified version of the OCEBM levels of evidence table, to provide a description of robustness. The breadth of the scoping approach meant that the depth of critical appraisal was limited, potentially leading to biases unrecognized by this scoping review. Third, though multiple high-quality databases were searched, unpublished or non-English studies may have been missed, and publication bias likely influenced the mapped evidence, as most of the included reports (particularly regarding CPV-2) described positive outcomes. Finally, data extraction was in some cases limited by incomplete reporting in some studies, mostly regarding diagnostic confirmation methods, dosages, exact treatment protocols, and possible adverse effects.

### 4.3 Specific findings by pathogen

The effects of IgY immunotherapy on CPV-2 infections was more thoroughly researched compared to other canine/feline pathogens, and according to studies reviewed herein, IgY was administered orally, nasally, and intravenously, the latter of which seemed to be the most effective route. IgY administration in CPV-2 infections generally led to improvements in clinical signs, faster recovery, enhanced leukocyte counts and hematological indices and reduced fecal viral load. A dose-dependent response was observed in 2 different studies, disease severity affected treatment efficacy in 1 trial, and 1 study inhibited clinical symptoms using IgY prophylactically. Many commercial IgY-containing anti-parvovirus products are available and widely used in veterinary medicine, including oral tablets such as Parvoguard™ and Parvocare™, and liquid suspension Canglob® P (which can be used intravenously, intramuscularly, or subcutaneously). Arguably, CPV-2 was one of the most successfully treated diseases by IgY immunotherapy, with Canglob® P becoming a routine part of therapy in refractory cases, but other disease such as CDV and ICH can also be treated by IgY products. Canglob® D Forte is a liquid suspension containing heterologous IgY against CDV, and Canglob® DHLaPPi contains IgY against CDV, CAV-1, CPV-2, canine infectious laryngotracheitis, and canine parainfluenza. These products can also be used as prophylactics or treatment options in immunocompromised or refractory cases, respectively.

The studies on the therapeutic effects of IgY on FIV/FeLV/FIP reviewed herein were conducted using a non-specific IgY-containing product. The non-specific IgY improved hematological indices, clinical symptoms, quality of life and survival time; however, when compared to disease-specific IgY used against CPV-2/CDV/CAV-1, the therapeutic benefits of the latter were much more pronounced. The evidence for the therapeutic effects of IgY in feline viral infections was generally weak, with most studies being case series and case reports, and only one non-randomized controlled trial, which lacked a true positive control group, but did study the effects of IgY discontinuation on symptoms recurrence. A non-specific product can theoretically be beneficial in FIV/FeLV, particularly when considering the threat of secondary opportunistic infections (bacterial/fungal) due to immunosuppression, but so far, the evidence was not strongly convincing; furthermore, this palliative effect could not be considered a cure, as it did not neutralize the virus. Perhaps further research should be conducted on specific anti-FIV/FeLV IgY products designed to neutralize the retroviruses and alleviate viremia.

### 4.4 Specific versus non-specific IgY

Based on the available literature, IgY-based immunotherapy specific to the target pathogen seemed to be more effective, particularly in the case of CPV-2 infections, which had shown the best response to anti-parvovirus IgY solutions. Non-specific IgY preparations were used to treat FIV/FeLV/FIP without strong efficacy. It was difficult to determine whether the ineffectiveness of non-specific IgY treatment in feline viral infections was due to the product or poor study design and limited sample size. However, another non-specific IgY solution was also produced and used against CPV-2/CDV/CAV-1, which led to faster recovery (Ali et al., 2024), and as mentioned above, Canglob® DHLaPPi also contains IgY against a range of different pathogens, suggesting that perhaps as long as the method of IgY production is directed against viral particles rather than non-specific secondary pathogens, the resulting preparation is more efficacious.

### 4.5 Gaps and limitations of the evidence

One common flaw among most of the studies reviewed herein, was the lack of randomized clinical trials (RCTs), which was ranked as the highest level of evidence in our modified scoring system. Only one study out of 14 mentioned randomized allocation of participants. Most of the studies were small non-randomized trials (*n* = 6) and the remainder were either case series (*n* = 3) or case reports (*n* = 3) and did not feature control groups. Sample sizes were generally small and ≤10, and only 5 studies featured ≥15 participants. OCEMB levels of evidence scores generally ranged from B-D. lack of blinding, randomized allocation of subjects, and true control groups in most trials, which was necessary to establish efficacy of the treatment regimen, signaled risk of bias. Hopefully as this method of immunotherapy becomes more widespread, studies with more robust methodology and stronger levels of evidence will emerge.

Evidence related to the therapeutic benefits of IgY in feline viral infections entirely came from the same core research group led by T.D. Supeanu and A. Supeanu and had not been independently replicated by other researchers. The IgY used in feline trials was non-specific, did not target viral particles, and was used to treat against a range of viral infections (FIV/FeLV/FIP), which may have limited its efficacy. Feline diseases have also been largely neglected when it comes to commercial IgY-containing products. Feline panleukopenia virus (FPV) is a parvovirus like CPV-2, from which CPV-2 evolved. The pathogenesis of FPV in cats and CPV-2 in dogs are very similar, with FPV also causing diarrhea due to its replication in rapidly dividing cells of intestinal crypts. Like CPV-2, FPV is also transmitted via the oral-fecal route and distributed via viremia (Parrish, 1995; Truyen et al., 2009). The remarkable similarities between FPV and CPV-2 and their taxonomic relatedness provides ample grounds for future therapeutic trials focused on specific anti-FPV IgY as main or adjunct treatment.

There are many protocols for IgY production, some which were explained at the beginning of this scoping review, but the lack of standardization for IgY production protocols, as well as its effective dosage and administration method, led to researchers experimenting with different routes of administration and production protocols, which added more difficulty to the comparison of IgY-immunotherapy efficacy across different trials. In addition, some studies had reported their therapeutic protocols, IgY production pipeline and diagnostic confirmation methods poorly, making reporting bias more likely. It must also be considered that most studies on CPV-2 reported positive results, so publication bias is possible, in additions to the fact that small trials are prone to exaggerate treatment effects.

### 4.6 Practical implications

IgY-based immunotherapy has been used as adjunct treatment against many different pathogens with varying levels of success. Barring any allergic reactions to egg proteins (which had not been recorded in any of the studies reviewed herein), the treatment was generally considered safe. As stated before, IgY treatments are a cheap, fast, and effective alternative solution to anti-viral and antibiotic (anti-bacterial) drugs, the former of which is only sometimes effective (depending on the method of virus replication), and the latter of which regularly causes antibiotic resistance, leading to hard-to-treat superbugs. IgY can be used both as non-specific palliative treatment (such as the studies on FIV/FeLV/FIP treatment), and as a neutralizing agent against specific pathogens (as in the studies on CPV-2/CAV-1/CDV treatment).

IgY has been suggested to facilitate passive immunization of patients by mainly 3 different mechanisms: 1) agglutination of pathogens leading to their immobilization and removal from the gut, 2) adherence-blockade and inhibition of pathogen adhesion thus impairing their function, 3) opsonization followed by phagocytosis and increased phagocytic efficiency (Abbas et al., 2019). Whether all or some of these mechanisms are responsible for the passive immunization attributed to IgY-based immunotherapy in the studies reviewed herein is a question that warrants further research, but it is reasonable to assume that in the cases where such protection was achieved, some form of pathogen neutralization mediated by IgY most likely took place.

In conclusion, IgY-based immunotherapy is a still developing subject of study, and based on the available literature, it appeared to be a promising alternative/adjunct form of therapy. It was well tolerated by patients and naturally lacked the complications and adverse reactions associated with antibiotics such as the development of antimicrobial resistance and toxic systemic effects, and contrary to antivirals, it could neutralize virus antigens in systemic blood circulation. It could be produced rapidly in copious amounts at a low cost, while causing the least amount of discomfort for the immunized hens. For diseases which include viremia/septicemia/secondary opportunistic infections as a main part of their pathogenesis, IgY is worth considering as a therapeutic option. However, large double-blind RCTs are still needed to evaluate long-term safety and immunogenicity of IgY products, as well as the most effective route of administration and dosage. Moreover, the application of IgY-based immunotherapy and commercial IgY products in feline medicine is still lacking, and future research should focus on producing IgY specific to feline pathogens such as FIV/FeLV/FIP/FPV or replicating the results of non-specific IgY (Supeanu studies) with more robust experimental designs. Only 1 trial studied the prophylactic effects of IgY, and considering the success of that study, the use of IgY to prevent disease in at-risk individuals is worth exploring. Better reporting standards in diagnostic confirmation methods, adverse effects and therapeutic protocols can greatly enhance our understanding of the role IgY could play against viral infections. Considering the relative ease and safety of pathogen-specific IgY production, it is expected that in the future, more canine and feline infectious diseases will have commercial, pathogen-specific IgY products, readily available to be used by veterinarians as adjunct/supportive treatment options for small animal patients.

### Ethical statement

This manuscript possess no animal and human experimentations/blood/tissue samples.

## CRediT authorship contribution statement

**Kamyar Madani:** Writing – review & editing, Writing – original draft, Visualization, Software, Methodology, Investigation, Formal analysis, Data curation, Conceptualization. **Nima Neyestani:** Writing – review & editing, Writing – original draft, Validation, Software, Methodology, Investigation, Formal analysis, Conceptualization. **Jalil Mehrzad:** Writing – review & editing, Writing – original draft, Visualization, Validation, Supervision, Software, Resources, Project administration, Methodology, Investigation, Funding acquisition, Formal analysis, Data curation, Conceptualization. **Darioush Shirani:** Writing – review & editing, Validation, Methodology, Investigation, Data curation. **Niloofar Zarifian:** Writing – review & editing, Software, Methodology, Investigation, Formal analysis.

## Declaration of competing interest

The authors declare that they have no competing interests.
